# Crime and punishment in times of pandemics

**DOI:** 10.1007/s10657-021-09720-7

**Published:** 2021-12-07

**Authors:** Roee Sarel

**Affiliations:** Institute of Law and Economics, University of Hamburg, Johnsallee 35, D-20148 Hamburg, Germany

**Keywords:** Deterrence, Pandemic, Covid-19, Uncertainty, Crisis, K42, K14, D9, D81, I00

## Abstract

How should we think about crime deterrence in times of pandemics? The economic analysis of crime tells us that potential offenders will compare the costs and the benefits from crime and from innocence and then choose whichever option is more profitable. We must therefore ask ourselves how this comparison is affected by the outbreak of a pandemic and the policy changes which may accompany it, such as governmental restrictions, social distancing, and responses to economic crises. Using insights from law and economics, this paper investigates how the various components in the cost-benefit analysis of crime might change during a pandemic, focusing on Covid-19 as a test case. Building on classical theoretical models, existing empirical evidence, and behavioral aspects, the analysis reveals that there are many potentially countervailing effects on crime deterrence. The paper thus highlights the need to carefully consider which aspects are applicable given the circumstances of the pandemic, as whether crime deterrence will increase or decrease should depend on the strength of the effects at play.

## Introduction

Predicting what would happen to crime during a pandemic, such as the one caused by the outbreak of Covid-19, is a challenging task. On the one hand, there are obvious factors that should *drive down* crime during a pandemic. For instance, the enforcement of governmental restrictions (e.g. lockdowns and quarantines), typically requires increased police presence in the streets, thereby discouraging individuals from committing crimes such as burglaries. On the other hand, other conspicuous factors may *drive up* criminal behavior. For instance, if restrictions hinder the ability to earn a lawful income, individuals might turn to crime as the only viable alternative.

In order to approach this topic analytically, one must rely on a structured framework that isolates the determinants of crime into different components and examines what happens to each component when a pandemic erupts. Luckily, such a framework is readily available in the economic analysis of crime. The economic analysis of crime assumes that individuals are rational and therefore decide whether or not to commit crimes using a cost-benefit analysis, comparing (at least) two options: committing the crime (i.e. choosing to be “guilty”) and abstaining from crime (i.e. choosing to be “innocent”). In the simplest version of this analysis, which builds on the canonical model by Becker (1968), individuals mainly consider three elements: the benefit from crime (“*b*”), the probability of being apprehended and punished (“*p*”), and the severity of the punishment (“*s*”). Individuals commit the crime if and only if the benefit from doing so exceeds the expected sanction (i.e. if $b>p*s$).^1^ Over time, this simple framework has been generalized to encompass a more elaborate set of choices. In particular, scholars have recognized that either option faced by individuals (crime or innocence) can entail both a cost and a benefit (see, e.g., Polinsky & Shavell 2000, 2007; Rizzolli & Stanca, 2012; Sarel 2018). Each of these may relate to elements that are either monetary (e.g. value of property stolen or a monetary fine) or non-monetary (e.g. subjective utility from hurting others or disutility from prison). Breaking down these elements is a first logical step for assessing how crime deterrence might be influenced by a pandemic.

A second step can be taken by looking into the vast knowledge accumulated in the empirical (and experimental) research on crime deterrence. The empirical literature confirms some, but not all, of the theoretical predictions of models *à la* Becker (1968), and also adds further insights closely related to pandemics. Among others, the literature highlights the tradeoff between *p* and *s*; the relevance of factors such as uncertainty, risk, time preferences, judicial errors, and behavioral influences (e.g. loss aversion and hyperbolic discounting); and the existence of long-term effects. This paper takes such a two-step approach and considers both purely-theoretical and empirically-informed predictions, thereby constructing a detailed thought exercise on how crime might be affected by pandemics. The lion’s share focuses on Covid-19, but the insights are general in nature.

The main insight arising from the analysis is that multiple countervailing effects may all play a part simultaneously during a pandemic, so that increases and decreases in crime are both plausible outcomes. Consequently, one must proceed with caution and avoid the temptation to declare an all-encompassing conclusion on how crime will change. Instead, the strength of the effects in play must be given proper attention.

The goal of this paper is thus to provide a list of possible channels through which a pandemic might affect crime. Such a list should be useful for both researchers and policymakers: for researcher, the effects laid out in the paper can help inspire theoretical predictions for specific cases of interest, which can then be tested using empirical research. In other words, the theoretical framework in the paper can help in generating new hypotheses. For policymakers, the paper can assist in the process of spotting different countervailing influences, which might otherwise be neglected. For instance, policymakers may find it easy to spot the more salient effects of the pandemic, such as the increase in unemployment (leading to a lower opportunity cost of crime) or in police presence in the streets during lockdowns (leading to higher probability of apprehension), but then neglect less conspicuous effects, such as the influence of loss aversion (e.g. when stealing is perceived as a recovery from monetary losses that are incurred due to the pandemic). Although the effects considered in the paper are numerous, their size is likely to be unequal and may well depend on factors such as the crime type, location, and market conditions. Hence, policymakers should keep in mind that in order to determine which of the effects described herein is economically important, one still needs to conduct an empirical analysis based on evidence that allows to capture and contrast the relevant factors.

The contribution of the paper is twofold. First, it lays out a framework for analyzing how crime deterrence might be affected by a pandemic, based on insights from theoretical and behavioral law and economics. This seems particularly useful for understanding the repercussions of the Covid-19 pandemic on (different types of) crime. In particular, while there is already some emerging empirical literature on the effects of Covid-19 on crime in specific areas (e.g. Abrams, 2021; Gerell et al., 2020; Calderon-Anyosa and Kaufman, 2020), as well as some theoretical discussions on compliance to Covid-19 mitigation strategies (e.g. Niavis et al., 2021; Elm & Sarel, 2021) and some other preliminary aspects of Covid-19 and crime (see, e.g., Skolnik, 2020; Miller & Blumstein, 2020), this paper provides a more comprehensive and generalized analysis by mapping various effects that may occur during a pandemic into the framework of crime-deterrence models. Second, my analysis of how loss aversion may affect crime during a pandemic contributes to the emerging literature on the behavioral economics of crime (see, e.g., Van Winden & Ash, 2012).

The rest of the paper is organized as follows: Part 2 reviews commonalities shared by pandemics that relate to crime deterrence. Part 3 considers separately how each component in the cost-benefit analysis of crime might be affected by a pandemic, adopting a simple Becker-style model of deterrence. Part 4 extends the discussion by considering additional effects identified in the empirical and behavioral literature. Part 5 concludes.

## What do pandemics have in common?

In the last two centuries, the world has been struck with a series of pandemics. Outbursts of diseases such as Cholera, Influenza, HIV/AIDS, SARS, and MERS have all led to a large number of infected people and many millions of deaths.^2^ The most conspicuous pandemic in recent history (prior to Covid-19) is perhaps the “Spanish Flu” of 1918–1920, which infected over a third of the world’s population and resulted in the death of over 40 million people (see, e.g., Barro et al., 2020). Yet, no pandemic has received so much global attention in real time as “Covid-19”—a name given to a respiratory disease, which began spreading across the globe in late 2019 and early 2020. This disease, which is caused by the “novel Coronavirus” (a.k.a. SARS-COVID-2), stands out as being highly transmissible and can be passed on even by individuals who do not show any symptoms.^3^ The Covid-19 pandemic originated in China but has struck the hardest in the US, Russia, Brazil, Peru, and several European countries.^4^

Pandemics share some commonalities as far as crime-related effects are concerned. First, a pandemic often leads to a financial crisis with both short-term and long-term negative consequences (see Jordà et al., 2020, for an overview). For instance, the Covid-19 pandemic was accompanied by a major downturn in many financial markets, massive increases in unemployment, and economic losses estimated in the range of trillions of US dollars (see, e.g., Fernandes, 2020; Barrero et al., 2020). As crime intuitively relates to the state of the economy in more than one way (each of which is discussed below in further details), the economic implications of a pandemic are clearly important for the question at hand

Second, pandemics all lead to rapid mortalities and other negative health consequences. Deaths are likely to have a direct effect on both the ability and profitability of committing certain crimes, as well as indirect effects, due to adaptations that society must take in response. For instance, if a certain population is struck harder than others (such as the older population, in the case of Covid-19), then any activities related to that segment of the population may change in ways that affect tangent criminal activities.

Third, pandemics instigate changes in public policy that are aimed to combat the disease. Apart from obvious responses (e.g. investing in medical R&D in search of vaccines), pandemics induce various forms of governmental restrictions on public life. Covid-19 provides a prominent example of such restrictions: in early 2020, as the world began to realize the severity of the disease, governments started implementing extreme measures targeted at “flattening the curve” of the disease’s progress. These measures include, among else, social distancing rules, quarantines, closing of state borders, and temporary shutdowns of some business activities (see, e.g., Teichman & Underhill, 2021; Elm & Sarel, 2021). Countries diverge in their approaches to handling the crisis, applying varying degrees of strictness when imposing and relaxing restrictions.^5^ Nonetheless, restrictions have clearly led to a drastic change in the daily life of many people.^6^

As of December 2021, the Covid-19 pandemic is still ongoing. However, there exists already some (mixed) empirical evidence on how crime rates have changed following the disease’s outbreak. In the early days of the pandemic, some studies found a substantial decrease in the overall level of crime in some cities, e.g. in San-Fransisco, California and in Oakland, New Zealand (see Stickle & Felson, 2020, for an overview). At the same time, other studies found mixed evidence of decreases, increases, and no effects at all—in particular when looking at individual crime types. For instance, evidence from Queensland, Australia showed a decrease in some property crimes (shop thefts, other thefts, and credit-card frauds) but no change in others, and no differences in violent crime (see Payne & Morgan, 2020a, b). In Sweden, there was evidence of a decrease in total crime, violent crime, and some property crimes (pickpocketing and burglary) (Gerell et al., 2020). In Los Angeles and Minneapolis, there have been decreases in violent crime.^7^ In a study on 25 U.S cities, Abrams (2021) found a decrease in drug crimes, theft, residential burglaries, and various violent crimes; no decrease for homicides and shooting; and an increase in non-residential burglaries and car thefts. Later studies, which focused on the effects of lockdowns (rather than the pandemic per se), similarly yielded mixed evidence. For instance, homicide rates in Peru decreased during a lockdown (Calderon-Anyosa & Kaufman, 2020); crime in Chicago was more frequent for domestic violence but less frequent for child violence (McLay, 2021),^8^ and shootings in New York City increased following stay-at-home orders (Kim & Phillips, 2021).

Explaining these mixed findings requires an understanding of the mechanisms at play. Yet, it is difficult to disentangle which pandemic-related elements are driving these changes in crime, including whether the effect stems from the disease itself or from the governmental policies that follow. Hence, the next section below approaches the question from a theoretical perspective, thereby isolating key elements that typically determine crime deterrence. For brevity reasons, the analysis abstracts away from the heterogeneity in governmental policies and instead takes a dichotomous comparison of pandemic vs. non-pandemic times. With a slight abuse of language, these will henceforth be referred to as “pandemic times” and “stable times”.

## Basic theoretical predictions

### Theoretical framework

Consider a Becker-style model of deterrence, where individuals face a dichotomous choice between crime and innocence.^9^ Formally, suppose that an individual j must decide between (1) committing the crime and becoming Guilty $(j=G)$ and (2) abstaining from crime and remaining Innocent $(j=I)$. Denoting the expected cost of each option by $E(c_j)$ and the expected benefit from each option by $E(b_j)$, the individual commits the crime if and only if the following condition is fulfilled:

$$\begin{aligned} E(b_G)-E(c_G)>E(b_I)-E(c_I) \end{aligned}$$

where the left hand side, $E(b_G)-E(c_G )$, marks the net utility from crime and the right hand side, $E(b_I)-E(c_I)$, marks the net utility from innocence, i.e. the opportunity cost of crime.

Some of the elements in this framework are straightforward. For instance, the benefit from committing a bank robbery directly translates into clear monetary terms (the value of the loot taken during the robbery). Similarly, the expected cost of committing a crime clearly includes the expected sanction, $p_G*s_G.$^10^ However, things become more complex if one adopts more elaborate assumptions. As one example, the defendant might face uncertainty regarding the probability of conviction or the penalty size, for instance, because of asymmetric information (see, e.g., Buechel et al., 2020) or because the level of incriminating evidence observed by the judge includes some stochastic component (see, e.g., Sarel, 2018). This then complicates the analysis. Moreover, some other elements are far more difficult to estimate. Consider, for example, the benefit from remaining innocent. What do individuals gain exactly from abstaining from crime? Obviously, if crime prevents one from earning a lawful salary (e.g. if crime commission is time-consuming, so that one cannot dedicate time for work) then the forgone salary constitutes an opportunity cost (see, e.g., Pelletan, 2020). Furthermore, if one ends up in prison and is incapacitated, future lawful salary is lost. However, one can think of additional benefits from innocence, such as subjective satisfaction from exerting self-control (see, e.g., Kokkoris et al., 2019).^11^ Similarly, the costs associated with innocence are also somewhat vague. The most notable factor identified in the literature is the prospect of wrongful convictions, which constitutes an implicit penalty for staying innocent.^12^ Presumably, one can also come up with other potential costs as well.^13^ To avoid confusion, the analysis below first assumes that potential offenders face fairly simple and identifiable elements. Thereafter, Part 4 extends the discussion in some additional directions.

### Effects on the net utility from crime

#### Benefit from crime

The benefit from crime is, of course, crime-specific. Consider, for example, *property crimes*, such as burglary, larceny, auto theft, or shoplifting, all of which yield some kind of monetary reward to the perpetrator. At first glance, one might think that the value of the reward is independent of pandemics, as stealing one dollar in stable times is just as profitable as doing so during a pandemic. Yet, some changes brought on by a pandemic might invalidate this initial thought.

First, if governments shut down borders, this hinders international trade in a way that can affect the profits made through criminal activity. Consider trade in stolen goods, such as cars: in stable times, criminals can ship out goods abroad and then (re)sell them on the black market without any frictions. Conversely, the shutdown of borders impedes such crimes or substantially increases the transactions costs incurred for carrying out the trade. Consequently, profits from crime will decrease (for a similar example regarding the sale of artworks, see Nicita & Rizzolli, 2009).^14^

Second, in a similar fashion, the economic turmoil might lead to changes in the value of goods, which in turn also makes stealing less (or more) profitable. As one example (from stable times), there is some empirical evidence from the Czech Republic demonstrating that the rate of metal theft depends on the price of metal (Brabenec & Montag, 2018). Hence, a potential offender who targets a good for theft may be more willing to commit the crime if that good becomes more expensive.^15^

Next, consider instead the benefit from *violent crimes,* such as murder, manslaughter, or aggravated assault. These crimes might yield a monetary benefit (e.g. if the victim somehow gets in the way of the offenders’ gaining of profits), but may also entail a subjective benefit.^16^ For instance, in domestic violence, spouses may quarrel out of anger and spite—even though the violence yields no monetary benefits (or even hinder such benefits).^17^

Again, at first glance, there is little reason to suspect that a pandemic will affect such benefits, and again this might be incorrect. First, if households are forced into quarantine, domestic violence might increase, as the mutual anger between spouses likely amplifies. A perpetrator then gains more subjective utility from hurting a spouse violently. Some studies indicate that indeed domestic violence increased during Covid-19 (see, e.g., Leslie & Wilson, 2020; McLay, 2021), supporting this possibility, but the evidence on this point is, again, mixed (see Nix & Richards, 2021).

Second, as the pandemic might tighten the availability of resources, e.g. because supply chains are broken, competition may lead to violence. As one anecdotal example, in the early stages of the Covid-19 pandemic, supermarkets were running out of some basic products, such as toilet paper (Kirk & Rifkin, 2020). As a result, violent incidents were documented in which customers fought one another to secure the limited products for themselves (see, e.g., Team & Manderson, 2020).

As a different example, consider *drug crimes*, including both trafficking and consumption. When people are quarantined, the demand for drugs may increase, as individuals search for any form of escapism. At the same time, the supply of drugs may decrease due to supply-chain constraints (for an overview of the impact of Covid-19 on the illicit drugs market, see, e.g., Barratt & Aldridge, 2020). The increase in demand and decrease in supply should jointly translate into a higher price for drugs, making trafficking more profitable. Conversely, drug consumption may yield either higher benefits (those which drive the increase in demand) or lower benefits (given the increase in the price).

Otherwise, for some other crime types, the prediction seems more straightforward. For instance, if a pandemic causes people to stay home and work remotely using computers, this should lead to a higher benefit from *cyber crime*. And indeed, the existing evidence from the Covid-19 pandemic mostly indicate an increase in such crimes, thereby supporting this conjecture.^18^

#### Expected sanction when committing a crime

In order to analyze how a pandemic—and in particular how governmental interventions that accompany it—might affect the expected sanction of *guilty* individuals, both the probability of being punished $p_G$ and the sanction size $s_G$ must be taken into account. When thinking about the probability $p_G$, it is important to note that it typically consists of a series of conditional probabilities, including the probability of detection, probability of arrest (conditional on detection), probability of conviction (conditional on arrest), and probability of reversing a conviction on appeal. How do these change during a pandemic? A first obvious effect mentioned above is the fact that the probability of being caught for a crime might increase due to enhanced police presence in the streets. This seems surely plausible for crimes for which one can get caught “red-handed”, such as burglary, theft, arson, or drug-trafficking on the streets. Yet, the effect of growth in police presence might be offset by officers who adjust their behavior in a time of a pandemic. For instance, there is some evidence of strain due to fear of Covid-19 infections among police officers (Frenkel et al., 2021) as well as evidence of general anxiety (see, e.g, Laufs & Waseem, 2020; De Camargo, 2021). If officers under-perform under these circumstances, criminals may expect a lower probability of apprehension in equilibrium. Such an effect may wary over time, as police forces reorganize and adopt processes that allow for better coping. However, such processes may also yield a shift in police priorities, inadvertently yielding a lower probability of apprehension for low-level crimes (for recent evidence, see Jennings & Perez, 2020). Furthermore, for some crimes, a detective’s work is the critical element of police work. If a pandemic causes a delay in forensics (see, e.g., Cattaneo, 2020; Moseley, 2020),^19^ police agility (see, e.g., Frenkel et al., 2021), or availability of witnesses (e.g if witnesses are sick), then the probability of being caught decreases compared to stable times. Additionally, when the police become constrained (e.g. because some police officers become sick), the quality of investigations might deteriorate, thereby mitigating the risk of being arrested.^20^

That said, a countervailing effect might emerge due to governmental measures. Consider, for example, Israel’s decision to track people’s location via their cellphone during Covid-19 in order to alert them if they were near a known infected person (see, e.g., Teichman & Underhill, 2021) for behavioral aspects of Israel’s policy). Such surveillance measures might make it extremely simple for the authorities to identify criminal activity in real-time as well as ex-post.

Additional aspects are crime reporting and the police’s response to reporting. On the one hand, victims and witnesses may be busy with pandemic-related issues and fail to pay attention when crimes are committed. This reduces the probability of being reported. On the other hand, people in quarantine may be less preoccupied compared to stable times and thus more willing to report.^21^ The police may be either faster or slower to respond, for similar reasons.

Next, consider the role of the criminal justice system in $p_G$: prosecutors, juries, trial judges, and appellate judges might all alter their behavior in various ways in response to a pandemic. For instance, during Covid-19, some courts decided to conduct virtual hearings via video conferencing (see, e.g., Baldwin et al., 2020). Intuitively, the angst that such hearings create for criminal trials is that the testimony would be less reliable or that the defendant’s right to cross-examine witnesses would be infringed (see Rowden et al., 2010; Rowden & Wallace, 2018, for a general discussion of such effects in remote testimony). Recent experimental evidence (not specifically related to courts) find, however, that there is no information loss when conducting a video conference compared to face-to-face meetings (Jabotinsky & Sarel, 2020). Thus, the switch to virtual trials might not matter much.

The same does not necessarily extend to sentencing, which relates to the severity of the sanction, $s_G$. In some countries (e.g. Australia and the UK), sentencing via video conferencing has already been implemented in stable times (Rowden & Wallace, 2018), leading to some controversies. Some have raised a concern that the use of video conferencing might make it psychologically easier for judges to impose harsher sentences (Rowden et al., 2010). Furthermore, virtual hearings imply that the judge does not get to observe the defendant’s family members in the courtroom, which may reduce compassion. Contrarily, video conferencing can reduce the court’s workload (Wallace et al., 2017), so that the judge can dedicate more time to each defendant. Possibly, this can lead a judge to be more empathic.

There are many additional pandemic-related factors (other than the switch to video conferencing), which may affect $s_G$. First, as prisons might become an “infectious beehive” at a time of a pandemic, judges might be reluctant to hand out prison sentences.^22^ If this is anticipated at the time of the crime commission, potential offenders effectively face a lower expected sanction.

Second, however, for the exact same reason, a prison sentence becomes implicitly harsher, as it entails a high risk of infection and of isolation. Some studies show that conditions inside the prison are relevant for the severity of the sanction (see, e.g., Katz et al., 2003), and again, if this is anticipated, individuals may fear going to prison more than usual.^23^

Third, for crimes punishable by a monetary fine, the same arguments made above with respect to monetary benefits apply. Here, on the one hand, changes in the price of goods might cause a depreciation in the currency, making the fine relatively less costly to pay. On the other hand, if there is a financial crisis, individuals might have a tougher time paying the fine, as (1) unemployment rates rise and (2) credit may be more difficult to get. Consequently, a pandemic might increase the share of so-called “judgment-proof” offenders (Shavell, 1986), who cannot afford to pay the fine (and, anticipating this, remain undeterred). In turn, this may force judges who care about deterrence to impose more prison sentences or to use some creative tools, such as “day fines” (see, e.g., Kantorowicz-Reznichenko, 2015).

Fourth, some aspects of $s_G$ may take informal forms, such as stigma (see, e.g., Mungan, 2017b). Namely, individuals considering whether to commit a crime may fear that a conviction would take a toll on their reputation, leading to either social outcasting or difficulties in finding a job.^24^ Stigma thus serves as an implicit penalty. The question is therefore whether stigma is stronger or weaker in times of a pandemic and here two opposite outcomes may emerge. Consider, for example, a person who decides to steal. On the one hand, society may be more forgiving towards a person who steals due to distress, e.g. because supplies have run out in the supermarkets. On the other hand, society may perceive stealing in a time when everyone is in need as extremely reprehensible, so that the stigma would be even stronger.

Fifth, an (obvious) cost of committing crimes during a pandemic is the fear of getting infected in the process of preparation for (or commission of) a crime. Presumably, those who would not hesitate to go out and commit a crime in stable times may change their mind when a pandemic is in the horizon.

Sixth, the administrative costs of committing crimes, such as the costs of acquiring a weapon or drugs, may be higher due to the disruption in supply chains caused by a pandemic. Similarly, crimes that require physical gathering in groups (e.g. conspiracies to commit murder)^25^ might be more difficult to coordinate during a lockdown. This, in turn, discourages crime commission.

Seventh, for some crimes, there is an implicit sanction in the form of vigilance on the part of victims (Smith & Vásquez, 2015). As victims may be more likely to remain indoors during a pandemic (either due to formal lockdowns or as a voluntary decision), potential criminals may fear being detected by victims. This may especially matter in areas that adopt “Stand Your Ground” policies, which enable victims to defend themselves by attacking the criminal without any need to first retreat (see, e.g., Yu, 2014; Gius, 2016). On the flip side, some locations may be more attractive as a target of crime commission, e.g. empty office buildings where there is little fear of vigilance.^26^

Summing up, even when considering only the net utility (expected benefits minus expected costs) from crime, there are already many possible countervailing effects.

### Effects on the net utility from innocence

#### Benefit of abstaining from crime

Analyzing the net utility from innocence requires considering similar types of factors as those considered above, as a pandemic changes not only the payoffs from crime but the payoffs from other activities.^27^ Consider, for starters, how the benefit of abstaining from crime might change during a pandemic. An obvious effect mentioned already is individuals’ ability to earn a lawful income through employment. The wage itself also plays an important role—if wages drop, then even those who are employed would have a higher temptation to switch to crime. In other words, when individuals cannot earn enough money through labor, the *relative* profitability of crime increases through the opportunity cost (for empirical evidence, see Entorf & Spengler, 2000; Gould et al., 2002).

As unemployment rates have skyrocketed due to Covid-19, one might expect crime to surge. However, as mentioned, the empirical findings thus far are highly mixed. This might be attributable to the countervailing effects described above, but also possibly to the governmental responses to the economic downturn. Namely, in response to the negative economic consequences of Covid-19, governments have implemented mitigating measures, such as a 2$ trillion relief package in the US and the “SURE” support package in the EU. Insofar that such measures are anticipated, the detrimental effects of unemployment to the net benefit from innocence are ameliorated.

A similar point can be made with respect to the closing down of schools during a pandemic and juvenile crime. Existing studies find that schooling can reduce crime, which can be explained also by the fact that teenagers in school have a high opportunity cost (falling behind on the study material).^28^ Then, the question simply becomes which steps (if at all) governments take to compensate for the loss of educational activities.

#### Expected sanction when abstaining from crime

Next, consider what happens to the cost of remaining innocent. Defining the costs of innocence is tricky and likely to be crime-specific. For instance, paying taxes might be the “cost of innocence” for tax evasion, but has no bearing on e.g. committing murder (while still paying taxes). Thus, the analysis in the existing literature typically focuses on explicit sanctions, such as wrongful convictions. Then, the more a person is likely to face a penalty when choosing “innocence”, the less profitable it is to choose that option.^29^

While there exists extensive research on wrongful convictions (see, e.g., Gould & Leo, 2010; Gross, 2016) predicting their effect on deterrence in a time of pandemics can be challenging. First, one must identify the causes of wrongful conviction and check how these change in times of a pandemic. Some causes are straightforward, such as witness misidentification, coerced confessions, misleading forensic evidence, or inadequate legal representation of defendants.^30^ The channels through which these could be affected have been partially discussed above. For instance, the video conferencing used in criminal cases might increase the probability of witness misidentification. Other channels are probably case-specific: a pandemic might make it more difficult for defendants, as well as for prisoners who seek to appeal a wrongful conviction, to gain access to proper legal counseling (see, e.g., Bošković & Nenadić, 2021).^31^ Conversely, as many lawyers may have difficulty getting clients at a time of a pandemic, the supply side of counseling hours may in fact increase, so that high-quality lawyers become cheaper and more available. In the case of Covid-19, the outbreak of the pandemic led to delays in many courts around the world (see Sourdin et al., 2020), which created a bottleneck for appeals. At the same time, some lawyers responded in innovative ways (e.g. by developing digital apps; see Cooper, 2020), which can mitigate the problem of access to justice.

There are also more complex channels of influence on wrongful convictions that are less straightforward. Consider, for example, the (fairly recent) theory in law and economics known as “compromise verdicts” (Lundberg, 2016; Sarel, 2018). In a nutshell, this theory states that judges who are uncertain of the defendant’s guilt may choose to balance their uncertainty through their sentencing discretion. As a result, defendants who seem guiltier will receive harsher sentences, and vice versa.^32^ What happens to uncertainty on the defendant’s guilt during a pandemic? One possibility is that in the midst of the pandemic-chaos, there is more noise, as not all evidence can be easily collected. Thus, judges may be less certain of the defendant’s guilt, so that they will more often (wrongfully) convict but impose a lower sentence. Another possibility is that prosecutors will offer more attractive plea bargains, to avoid the need of conducting a logistically complex trial. This will again result in more wrongful convictions, but lower penalties. The existing evidence on plea bargains during Covid-19 suggest that indeed plea bargains may be more lenient (Daftary-Kapur et al., 2021) but also that false confessions then increase (Wilford et al., 2021), in line with the latter possibility.

Summing up, the net utility from innocence is also subject to potentially countervailing effects, making predictions quite challenging.

## Empirically informed predictions

In order to get a comprehensive picture of the determinants of crime deterrence during a pandemic, it is important to go beyond the theoretical literature and consider also the advances that have been made in the study of deterrence in recent decades. In particular, one should take into account effects identified in the vast empirical and experimental literature (see Chalfin & McCrary, 2017; Engel, 2016, for an overview) that might be more (or less) likely to arise during a pandemic.

### Probability of being punished versus the size of the sanction

A first point relates to the difference in the elasticity of crime with respect to the probability of apprehension, on one hand, and the penalty, on the other hand. Although the literature provides highly mixed findings, some relevant points can be identified.

First, the empirical literature mostly shows that individuals are more responsive to changes in probabilities than changes in the sanction size. For instance, crime rates seem to be more affected by changes in the number of police officers or police tactics rather than reforms which increase the penalty. Yet, the “credibility revolution” in economics, which strives to identify causal links instead of relying on correlations alone, has brought to light various studies that do find a significant deterrent effect of penalties on both crime rates and recidivism (see, e.g., Bhuller et al., 2020; Eren & Mocan, 2017). Furthermore, there exist some experimental evidence suggesting that changes in the penalty size may even have a stronger effect than changes in probabilities (see Friesen, 2012). One explanation for the divergence, pointed out by Mungan (2017a), is that observational studies (e.g. in criminology) measure aggregate crime rates whereas economic experiments focus on a treatment effect. The two may then diverge because of reasons such as the role of repeat offenders, which are not always considered in experiments.^33^

The discussion in the previous part highlights the fact that both the probability of being sanctioned and the sanction size (either for innocents or guilty individuals) may change during a pandemic. Moreover, countervailing effects were pointed out for each element. Thus, a first general insight when considering the empirical evidence is that some arguments should perhaps be given more weight than others—but it is not fully clear which ones. A possible way out of this ambiguity can be taken by focusing on the stream of literature that attempts to explain why the findings diverge. Some of the existing explanations highlight general equilibrium effects, where changing the sanction size might indirectly lead to changes in the probability of conviction because judges or prosecutors respond to the change in the sanctions (see, e.g., Andreoni, 1991; Lundberg, 2016; Sarel, 2018). Other explanations focus on how individuals respond to uncertainty more generally.

**Fig. 1:**
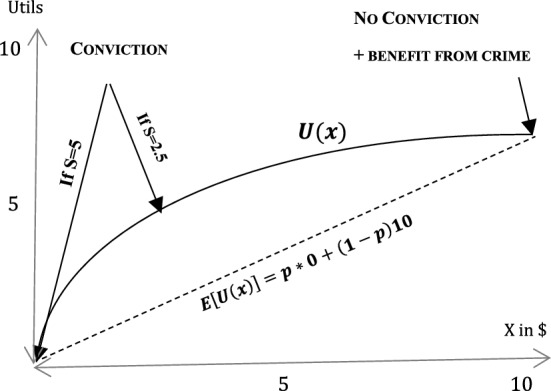
Illustration of risk aversion

A first aspect in this regard, already considered in Becker’s original model, is *risk aversion*, where a risk-averse individual gains higher utility from a safe payoff compared to a stochastic payoff that yields the same amount on expectation. To illustrate, consider an individual who gains utility from having money in the amount of *x* according to a utility function, *U*(*x*). More money yields a higher utility, $U^{'} (x)>0$, but the marginal benefit from more money decreases (e.g. because a rich person cares less about one additional dollar compared to a poor one), i.e. $U^{''}(x)\le 0$. Such a utility function has a concave form, as depicted in Fig. 1.

Suppose that the individual has 5$ in his bank account and is considering whether to steal another 5$, where a conviction means that the individual must give back the stolen money and is additionally penalized with a monetary fine of 5$ (bringing his overall wealth to zero). If the individual abstains from crime, he keeps 5$ and gets the utility from having this money, i.e. U(5). Conversely, committing the crime is conceptually equivalent to a lottery ticket paying 0$ with 50% probability and 10$ (=5$ owned plus 5$ stolen) with a probability of 50%. Note that the expected payoff is the same irrespective of whether the crime is committed: it is 5$ (for certain) when abstaining and 0.5 * 0 + 0.5 * 10 = 5$ when committing the crime. However, the expected utility, represented by the dashed line in the figure, is $$EU=0.5U(0)+0.5U(10)$$.^34^ As can be seen, the concave function (due to risk-aversion) means that the utility from abstaining (*U*(5), i.e. 5 for certain) is higher than the lottery when committing a crime (*EU*(5)) so that the crime will not be committed. When a pandemic erupts, uncertainty might increase. Considering the same example, this may mean, for instance, that the individual cannot gain 5$ for sure when stealing (e.g. because people may be at home so that stealing becomes infeasible). As a result, the expected utility from stealing will decrease, leading to more deterrence at the margins. Naturally, the exact opposite can also occur: the existing wealth of 5$ may no longer be guaranteed because of unemployment so that the utility from innocence decreases. This example demonstrates that the transition from safe to risky might, by itself, trigger a change in behavior when individuals are risk-averse.

In Becker’s original model, risk aversion is partially responsible for one of the most controversial results for crime deterrence: if individuals are risk-averse, a social planner should set the probability of apprehension close to zero and the penalty infinitely high.^35^ The reason is that risk aversion makes it so that a severe sanction *s* alongside a low probability *p* is more effective for deterrence than a lenient sanction and a high probability, even if the expected sanction is the same in both options.^36^

**Fig. 2:**
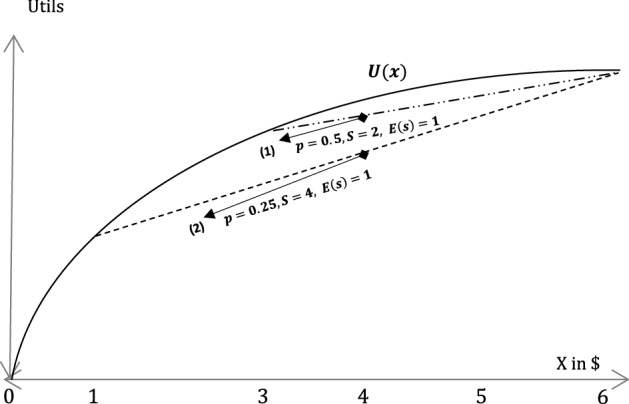
Expected sanctions with risk aversion

This is illustrated in Fig.  2: Suppose that an individual who already has 5$ (as in the previous example) can steal 1$ and that the social planner is trying to choose between two menus of sanctions:^37^

- Menu (1): $P=0.5, S=2$. The expected sanction is $0.5*2\$=1$.
- Menu (2): $P=0.25, S=4$. The expected sanction is $0.25*4\$=1$.

Menu (1) implies an expected utility on the upper dotted line (still below the utility function *U*(*x*)) whereas Menu (2) implies an expected utility on the lower dashed line.

As can be seen, Menu (2), where the sanction is higher but the probability is lower, generates less utility compared to Menu (1), although both menus yield the same expected sanction.

Relating back to pandemics—the question is then what exactly changes. Possibly, both the sanction size and the probability of conviction change (e.g. because judges respond to the uncertainty, as mentioned above). Individuals will then respond differently, depending on their degree of risk aversion. There is some evidence gathered in connection with the willingness to tolerate a risk of Covid-19 infection, which points at differences in the degree of risk aversion across different age groups and financial status (Kluwe-Schiavon et al., 2021). If this type of risk aversion carries over to the decision to commit a crime, one can perhaps use these parameters to identify groups that are more likely to commit a crime at the margins during a pandemic. Yet, a far more intriguing insight that can be gained here concerns behavioral effects (see below).

### Loss aversion and prospect theory

Behavioral law and economics can shed further light on how crime deterrence might be affected by a pandemic. Consider, for example, the well-established *prospect theory* (Kahneman & Tversky, 1979), which adds two key assumptions: First, individuals are assumed to be risk-averse when a payoff they receive is (subjectively) perceived as a gain but are risk-seeking when the payoff is perceived as a loss. Second, individuals are “loss averse”: so that they respond more starkly to losses compared to equally sized gains (typically described as “losses loom larger than gains”). A later refinement of the theory, known as “cumulative prospect theory” (Tversky & Kahneman, 1992), highlights that whether or not individuals perceive a cost as a loss depends on their (possibly subjective) benchmark, a.k.a “reference point”. Often, the reference point will be the status quo that exists prior to taking a decision—here, the decision to commit a crime. However, there may be other reference points as well (see Koop and Johnson 2012, for an overview), which is highly relevant for the issue of pandemics, as will be explained below.^38^

**Fig. 3:**
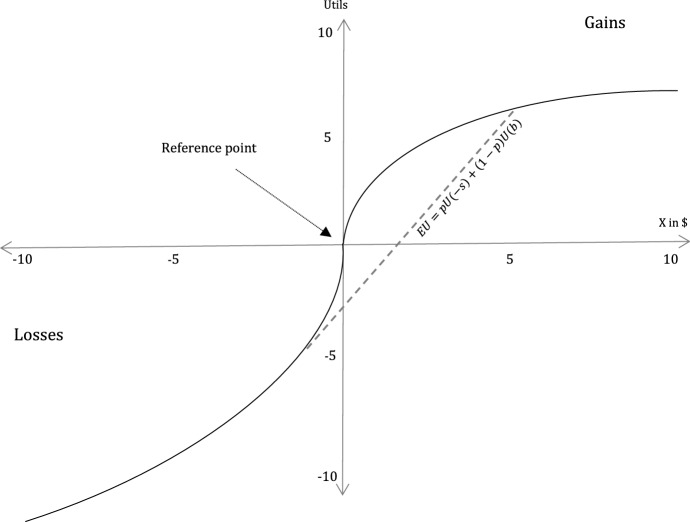
Prospect theory (stable times)

The existing literature which links prospect theory to crime deterrence has generally concluded that Becker’s model holds also if individuals are loss averse, with some exceptions (see Dhami & al Nowaihi, 2018; Rizzolli & Stanca, 2012; Nicita & Rizzolli, 2014).^39^ Yet, prospect theory holds some interesting implications for pandemics. For illustration purposes, Fig. 3 presents a standard utility function according to prospect theory.

Suppose that a person has no money at all, i.e. 0$, and must decide whether to commit a crime that yields a benefit *b* and entails a monetary sanction *s* that is imposed with probability *p*. The expected utility is thus $$EU =p*U(-s)+(1-p)*U(b)$$ as depicted by the dashed line. If the status quo serves as the reference point, any money earned through criminal activity is perceived as a gain, which lies in the upper-right quartile of the figure. It can be easily seen that this quartile is identical to the risk-aversion case considered above in Figs. 1 and 2.

Conversely, a monetary fine would place such a person with a negative balance, i.e. in the domain of losses. Note that the utility function is different in two ways compared to gains: it is convex (reflecting risk-seeking) and it has a steeper slope near the reference point (reflecting loss aversion). In this particular example, the general logic of the Becker model holds: if the probability of not being punished $(1-p)$ is sufficiently high, the utility from crime is higher than the utility from the status quo (as the upper-right end of the dashed line is above 0).

The question is thus what happens to this analysis when a pandemic erupts. While it may be challenging to consider all possible scenarios here, one option is that a pandemic causes people to perceive their status quo (prior to committing a crime) already as a loss. Particularly, if the status quo in stable times serves as the reference point, then calamities such as sudden unemployment, a financial crisis, and health insecurity cause stealing to be perceived as a recovery of lost money rather than as a gain.

**Fig. 4:**
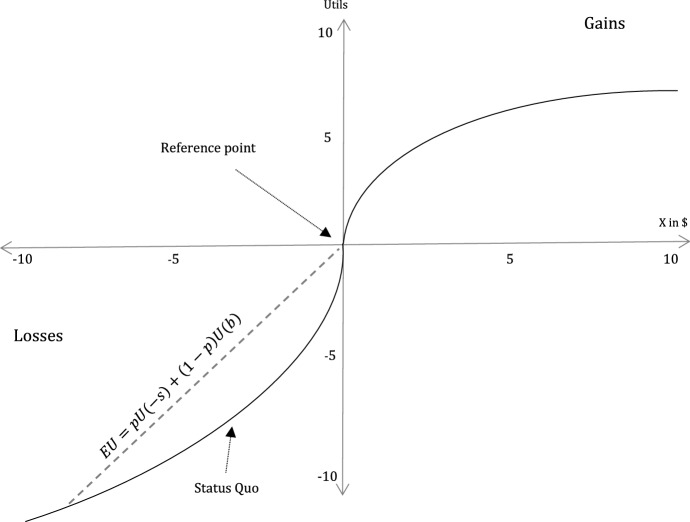
Prospect theory (pandemic; loss domain)

To emphasize the consequences, consider a slightly revised version of Fig. 3 in the form of Fig. 4. Suppose that the individual lost an amount of *b* when the pandemic started, e.g. due to medical bills or loss of salary. The status quo of that person yields the utility $U(-b)$ but, unlike the previous example, it is *not* the reference point.

Visually, the dashed line now lies completely above the utility function in the domain of losses and the vast majority of the line is above the status quo. What this means is that, ceteris paribus, crime commission becomes more likely compared to stable times. The intuition is simple: given that the individual is risk-preferring in the domain of losses, the fear of “a bit more loss” due to a potential penalty is not as frightening, whereas recovering what has already been lost starkly increases utility. In other words, people who already feel they are losing are willing to take a risky gamble and increase their loss, in the hope of avoiding the initial loss.

As a more concrete example, consider shoplifting during Covid-19. When the novel Coronavirus emerged in China in late 2019, and the U.S. was still untouched by the virus, stealing supplies from a store was plausibly perceived as a gain. Conversely, when supplies became scarce, individuals might have perceived *not having* them as a loss. Stealing thus became, ceteris paribus, more likely—as individuals felt that doing so mitigates a loss rather than generates a gain.^40^

However, a countervailing effect may emerge due to a related feature of prospect theory: the “certainty effect” (for recent experimental evidence in the context of crime, see, e.g., Pickett et al., 2020). This effect causes people to attribute higher weight to switches from uncertain to certain outcomes (e.g. from 90 to 100%), compared to equally sized switches between two uncertain outcomes (e.g. from 80 to 90%). Intuitively, for this reason, a certain sanction might be more deterring than a stochastic one (see, e.g., Johnson & Payne, 1986). But, for a similar reason, the status quo also becomes uncertain, e.g. because individuals are not sure if and when supplies will be available. A decline in certainty for the status quo option causes the initial loss to be stochastic, and an uncertain loss is then “less painful” than a certain one.

The main takeaway from this discussion is that behavioral effects further complicate the ability to make meaningful predictions on the effect of a pandemic on crime deterrence. However, insofar that individuals do indeed perceive themselves to be in the domain of losses during a pandemic, this has a policy implication for the tradeoff between the probability *p* and the sanction *s*. Namely, the opposite of risk-aversion holds: keeping the expected sanction fixed, it is no longer optimal to set the fine infinitely high and the probability of punishment very low, as individuals might tend to be more risk-seeking when a pandemic erupts.^41^

### Time preferences

Another relevant aspect, supported by both theory (e.g. Davis, 1988) and empirics (e.g. Lee & McCrary, 2017), is time preferences. Specifically, as there is usually a time difference between (1) the event in which the benefit from crime is attained and (2) the date in which a penalty is imposed (conditional on being convicted), the elements *b* and $$p*s$$ might not be directly additive. Instead, future payoffs are typically discounted by a factor lower than 1.^42^ Consequently, future penalties become less painful than immediate penalties. Discounting may occur for several reasons, including the time-value of money.^43^ For instance, consider two individuals, one who must pay a fine of 1000$ directly after committing the crime and another who must pay the same amount but only a year later. The latter individual is better-off, because he can make profits off of the (yet unpaid) fine, e.g. by investing the money and earning interest.

Some papers (e.g. Mastrobuoni & Rivers, 2016) go so far as to blame time preferences for the occurrence of crime, arguing that offenders are likely to be those with strong time-preferences, so that they prefer to get the (immediate) benefit from crime while heavily discounting future sanctions. The question is, once more, how time preferences relate to pandemics. One effect might be that a pandemic causes people to fear that they will die sooner than expected, e.g. because they get infected themselves; because they will not be able to get proper medical treatment for other problems if hospitals are overrun with infected patients; or because they will not be able to afford medical treatment due to the consequences of losing their job.^44^ As a result, people may discount the future more heavily—causing some individuals who would usually not commit a crime in stable times to commit a crime during a pandemic. On the other hand, the benefit from crime may also be heavily discounted for the same reason, e.g. when goods are stolen but cannot be consumed over time. Thus, once more, it is difficult to tell which effect will dominate—also because individuals might misestimate the probability of dying or getting sick.^45^

### What happens if higher sanctions cause crime to increase?

A different source of ambiguity, which has been identified in existing studies, is the effect of sanctions on deterrence. While the Becker model predicts that harsher sanctions will lead to more deterrence, this claim has been criticized on several fronts. For instance, it has been argued that longer prison sentences might lead to more crime in the long run, as prisoners spend their time learning how to become better criminals (so-called “school for crime”; see, e.g., Blumstein (1998) and Nguyen et al. (2017)). Other arguments focus on a ‘marginal deterrence’ argument: if individuals face more than two options (e.g. multiple crimes or different levels of crime-severity), then increasing the penalty to the maximum on all offenses will simply cause offenders to select the crime with the highest benefit. For instance, if consuming 100g and 10g of illegal drugs are both punishable by a maximal fine, then offenders will prefer the more problematic option (in this case, consuming more drugs) (Caulkins, 1993). Following a similar logic, raising the penalty on a specific crime might cause the individual to simply switch to a different crime, so that deterrence will not increase.^46^

There are several additional arguments for why an increased penalty may undermine deterrence. One argument focuses on psychological effects such as cognitive dissonance, where people might rationalize a high penalty as being associated with a large benefit from crime (which is otherwise underestimated; see, e.g., Dickens (1986)). Another argument diverts attention to a prisoner’s post-release prospect of earning a legal income: if serving more time in prison reduces the chances of finding a job afterwards, then the post-release opportunity cost of crime decreases (see, e.g., Pelletan, 2020).

The interesting question is then whether these arguments are more likely to be applicable during a pandemic. Considering each and every option here is out of scope for this paper, but some examples can again be highlighted. First, some crime opportunities that exist in stable times might disappear during a pandemic (e.g. because a business that could have been robbed is forced to close down). Thus, there are fewer illegal options to switch to if the penalty on another offense increases. This suggests that, ceteris paribus, an increase in the sanction is *less* likely to be detrimental to deterrence during a pandemic (compared to stable times). Conversely, it is possible that the pandemic creates new opportunities to commit crime, e.g. in the context of cyber crime (see Ma & McKinnon, 2021).^47^

Second, prison sentences might be shorter on expectancy during a pandemic, either because judges are less inclined to impose longer sentences (see above) or because governments respond to a pandemic by releasing some prisoners. For instance, Covid-19 has given birth to outcries calling for the reduction in overcrowding in prisons worldwide. And indeed, several countries have followed suit, setting policies for either temporary or permanent mass release of prisoners (see, e.g., Wang et al., 2020). Shorter sentences imply that there is less time to be “educated” in the “school for crime”, but also means that, ex-ante, individuals might discount prison sentences, anticipating their possible premature release.

Third, it is difficult to say whether cognitive dissonance behaves any different during a pandemic. It might be the case that individuals are less likely to interpret changes in the sanction as a proxy for the benefit, and instead attribute changes to the pandemic-related circumstances. Yet, the exact opposite is also somewhat plausible—where people would rationalize that any new actions taken by the government in a time of emergency probably reflect an urgent and real need to protect some valuable benefit from being appropriated.

### Incapacitation

The discussion thus far has focused on deterrence. However, if the purpose is to predict what happens to crime rates, incapacitation is important as well. Although deterrence and incapacitation are sometimes difficult to disentangle empirically (see, e.g., Levitt, 2004; Kessler & Levitt, 1999), the conceptual difference is clear: deterrence deals with the cost-benefit calculation of whether to commit a crime, whereas incapacitation reflects the (physical) inability to commit crimes, usually due to being in prison. As mentioned above, prisoners might be released en-masse during a pandemic. If the government takes proper precautions, e.g. by strictly monitoring the released prisoners, then the decrease in incapacitation will not matter much for crime rates. Conversely, if monitoring is imperfect, the released prisoners might be back to their old ways, especially if the job market prospects are slim.

A different type of incapacitation is lockdowns: individuals who are prevented from leaving their homes are, for most purposes, incapacitated. The degree of incapacitation depends, however, on how strictly lockdowns are enforced and the exact scope of restrictions. Still, this incapacitation is arguably a strong factor that can drive at least some forms of crime down.

### Expansion of existing crime categories

Another, more subtle, effect of a pandemic on crime might be how existing offenses are interpreted. For instance, following the Covid-19 pandemic, some behaviors that typically would not constitute a crime in stable times (e.g. coughing at another person) were suddenly be interpreted as “assault” in Canada (Skolnik, 2020). In terms of deterrence, this sort of effect is not a conceptual problem *per se*: as coughing turns into a (potentially) harmful activity when a pandemic erupts, there is no reason to assume that the usual calculation of costs versus benefits should not apply. Namely, a person considering whether to cough would now simply need to estimate the expected sanction for coughing and then decide whether the expected benefit is worth the risk. Thus, while a larger scope of behaviors that constitute an offense might lead to an increase in crime on a technical level, as long as deterrence works there is no particular reason to assume that crime rate would increase for this reason.

However, individuals might not know ex-ante which types of behaviors are suddenly criminalized, because often the change would occur through judicial interpretation of the existing criminal offenses rather than an explicit adoption of a new pandemic-specific crime. In this case, the ex-ante uncertainty might have the same effect as wrongful convictions (recall: these might decrease deterrence). Moreover, political economy considerations might lead decision-makers to prefer the expansion of existing crime categories over the creation of new crimes, as this avoids the legislative process and allows to create “pocket crimes” (see Yirong, 2019) that provide more leeway in the decision of who to punish. This desire might be especially strong when chaos emerges due to a pandemic. Hence, it is indeed unclear whether individuals can effectively estimate the exact expected sanction for these pocket crimes.

Furthermore, even if crime categories remain exactly the same, a pandemic can also provide a new incentive for committing crimes, that is otherwise absent in stable times. For instance, there is evidence that Covid-19 has led to an increase in hate crimes toward Asians as the group most associated with the source of the virus (Gover et al., 2020; Tessler et al., 2020). Conceptually, this can be perceived as an increase in the benefit from crime (offenders gaining pleasure from attacking this group) but what makes this slightly different is that the higher incentive to commit a crime is purely subjective. Such an effect can, theoretically, also emerge in any number of contexts, e.g. as a result of new conspiracy theories (for a discussion of the increase in the spread of such theories during the Covid-19 pandemic, see, e.g., Sternisko et al., 2020). In a pre-pandemic study, Jolley et al. (2019) find that the belief in conspiracy theories is a significant predictor of the intention to commit everyday crime. Jointly taken, this means that a pandemic can create various subjective incentives to commit crimes, which may contribute to an increase in the crime rate.

### Long-term effects

The last aspect worthy of mention is long-term effects. Suppose that a pandemic comes and goes, and the world returns to its daily routine. Should we then expect any lasting effect of a pandemic on crime? And if so, when should such an effect emerge? Again, there are many possibilities in this regard (see Ceccardi, 2020), but two interesting ones do stand out. The first relates back to prospect theory: if people’s reference point changes because of a pandemic, the cost-benefit calculation that follows will inevitably change as well. A particularly relevant change is intertwined with another well-established behavioral effect, known as “sticky defaults” (see, e.g. Almlöf & Bjuggren, 2019; Cappelletti et al., 2014): in a wide range of contexts, scholars have found that people tend to stick to the default they are given, even when doing so is costly. In the context at hand, many defaults that the population is used to in stable times suddenly change, as movement is restricted, social contact is prohibited, etc. If this new reality sinks in as the “new default”, then one would see less deviations from it even as time passes. However, what this means for crime is again unclear: any defaults that facilitate crime commission (e.g. avoiding going to the office, which facilitates burglaries into office buildings) might cause more crime in the long run, and vice versa. Thus, a first possible long term effect is thus simply the inertia of the outlined effects above.

A second, very different, long-term effect concerns child birth and abortions. Existing empirical evidence on crime in the US suggests that legalized abortions lead to a substantial decrease in crime rates in later years (see Donohue & Levitt, 2001, 2020). The reason is not very “politically correct”, but simple enough: unwanted children are at an elevated risk to be involved in crime when they grow up, so that allowing their mothers to terminate the pregnancy prevents them from growing up to become criminals. For obvious reasons, this argument has been a source of controversy.^48^ However, the effect of abortions can easily co-exist with the theoretical framework of this paper. For instance, unwanted children may be those who have a higher benefit from crime because they are poor; have lower opportunity costs due to their worse job prospects; or perceive their status quo to be in the domain of losses due to their rough childhood.

How do childbirth and abortions relate to a pandemic? When the Covid-19 pandemic emerged and lockdowns became omnipresent, predictions pointed at a possible “baby boom” in light of evidence from previous pandemics (see, e.g., Ullah et al., 2020). If a baby boom occurs, it is plausible that this might lead to more unwanted pregnancies than in stable times and, simultaneously, to less abortions. This can occur either because access to abortion clinics and hospitals might decrease during a pandemic (e.g. because some clinics are closed or because governments forbid abortions during a pandemic) or because pregnant women would be afraid to go through abortion due to fear of infection while being at the clinic.^49^

On the other hand, survey evidence from Italy indicate that a baby boom is not necessarily in-store for everyone, where some respondents even expressed a decision to abandon child-planning due to future economic difficulties (Micelli et al., 2020). Moreover, there are some evidence that Covid-19 can cause miscarriages (see, e.g., Hachem et al., 2020), which may reduce the overall effect. Furthermore, the link between abortions and crime might not hold for non-US countries, e.g. because the social welfare policies are different. Thus, the long-term effects are ambiguous as well (for an overview of the different predictions regarding child-birth and Covid-19, see Döring, 2020).

## Conclusion

“*I neither know nor think that I know*.”^50^

Analyzing how crime might respond when a pandemic erupts is a frustrating exercise. For each argument indicating that crime might decrease, there seems to be an equally plausible argument suggesting the opposite. Some of the arguments are straightforward, mostly because they cleanly map into the components of the Becker model (e.g. police behavior affecting the probability of apprehension or unemployment affecting the opportunity cost of crime). Yet, what the analysis reveals is that there are some more subtle countervailing effects, alongside starkly different effects (e.g. those related to behavioral theories). Notably, some of the effects discussed above are more plausible than others: for instance, the effect of police officers roaming the streets is likely to be a first-order effect, whereas changes such as video-conferencing of court hearings are likely to be second (or even third) order effects. Thus, each of the effects should clearly be weighted differently, depending on its relative importance.

Moreover, policymakers might also need to take into account the fact that the challenge of estimating the parameters of interest (e.g. probability of apprehension) is not unique to authorities, but applies to criminals as well. The optimal policy may then depend on the share of (un)informed criminals (see, e.g., Buechel et al., 2020), also keeping in mind that criminals may generally underestimate or overestimate the relevant probabilities (see Chopard & Obidzinski, 2021).

In the end, whether crime increases or decreases in any given area affected by a pandemic is an empirical question. However, one must be careful from falling victim to over-optimism regarding external validity: just because a pandemic “A” combined with government measures “B” caused crime of type “C” to change in area “D”, one cannot directly jump to the conclusion that this same will extend to other pandemics, measures, crime-types or areas. Instead, one must consider which effects are more likely to play a part, depending on the circumstances, and only then design a crime-reducing policy (if needed). Respectively, while this paper details a long chain of potential effects, its goal is not necessarily to provide a fully comprehensive guide to the effects at play, but rather to highlight how one should approach the topic. Instead, the paper points out the possible channels of influence, so that researchers and policymakers can be aware of the different potential effects.

As a final remark, it is worth mentioning that some of the effects may apply, with the appropriate adjustments, to other forms of crises other than pandemics. For instance, policemen may roam the streets on behalf of political leaders in order to suppress a political uprising or a civil war, and a surge in unemployment may occur also due to a financial crisis. Nonetheless, some of the effects seem to be unique to pandemics; most notably those relating to fears of infections and social distancing measures. Thus, while some of the insights are more general, others require a specific set of conditions to apply and thus depend on the particularity of the health situation.

## Data Availability

Not applicable

## Footnotes

1 Becker (1968) uses a slightly different notation: *f* for the sanction’s severity and *u* as a “portmanteau variable”, capturing income from other legal and illegal activities.

2 Cholera pandemics in the twentieth century include the “Sixth” (1899–1923) and “Seventh” (1961–1975) waves (previous waves occurred in the nineteenth century). Notable Influenza pandemics include the 1918 Spanish flu, the 1956–1957 Asian flu, and the 2009 Swine flu.

3 The degree to which asymptomatic patients are infectious is not yet fully clear (see, e.g., Skolnik, 2020).

4 According to the website Worldometers (https://www.worldometers.info/coronavirus/), there are more than 260 million Covid-19 cases worldwide, as of December 1, 2021.

5 For instance, Sweden largely allowed its economy to operate as usual, whereas Italy and Spain imposed strict lockdowns (see Andersen et al., 2020).

6 For instance, business have largely switched from an office-based workplace to a home-office approach, with video conferencing replacing face-to-face meetings (see, e.g., Jabotinsky & Sarel, 2020).

7 A recent study attributes the decrease in violent crime in Los Angeles and Indianapolis to the social distancing measures imposed by the authorities (Mohler et al., 2020).

8 An earlier study on crime in Chicago found even more mixed evidence, where effects seemed to vary by community (Campedelli et al., 2020).

9 Many papers follow this dichotomous approach (e.g. Png, 1986; Rizzolli & Stanca, 2012; Rizzolli, 2019) but some consider instead a continuous action space (e.g. Craswell & Calfee, 1986).

10 The subscript G refers to the expected sanction when the individual is guilty.

11 Kokkoris et al. (2019) find evidence of subjects gaining satisfaction from their decision to exert self-control and avoid temptations. Tittle and Botchkovar (2005) find that the degree of self-control is uncorrelated with fear from sanctions, which seems to further support the idea that satisfaction from self-control should be considered as a distinct benefit on the side of the payoff from innocence.

12 Classifying wrongful convictions as a cost of innocence is usually attributed to Png (1986). However, earlier papers (e.g Harris, 1970) make similar points. For a discussion of wrongful conviction and deterrence, see also Lando and Mungan (2017).

13 Note that in a simple dichotomous model, costs or benefits added to the RHS and LHS have symmetrical effects. However, in a more complex models of crime deterrence, the same cost (or benefit) may have different effects depending on which side of the inequality it lies. Specifically, in a behavioral model with loss aversion, there are additional perceived costs that are only incurred if one deviates from a reference point, which may be the payoff in the status quo (i.e. innocence) or some expectation of the payoff (for a recent example, see Feess & Sarel, 2021). Loss aversion is considered in further detail in Part 4.

14 Note that the increase in transaction costs may also occur for other related reasons, such as sudden changes in the exchange rates (e.g. when the Covid-19 pandemic erupted, the Yuan starkly devaluated compared to the USD; see Barua, 2020).

15 This effect is also known as the “rapacity effect” (see Dube & Vargas, 2013).

16 Some property crimes (e.g. vandalism) may also be centrally driven by subjective benefits, e.g. revenge against a rival.

17 Of course, it is also possible that the *objective* benefit from attacking a spouse increases during a lockdown. For instance, if the costs of supporting a two-person family become higher during a lockdown, then an attempt to remove another person can then be motivated by materialistic reasons and not only subjective ones.

18 Buil-Gil et al. (2021) find an increase in cyber crime in the UK during the early days of the pandemic (for slightly more mixed findings regarding the UK, see Hawdon et al., 2020). Kashif et al. (2020) also identify an increase in cyber crime, but use survey data. Some studies also raise concerns in light of Covid-19-specific cyber-scams (see, e.g. Collier et al., 2020; Lallie et al., 2021).

19 Moseley (2020) provides anecdotal examples from labs in the US on why delays in forensic occurred during Covid-19. These include staffing problems, decisions to re-purpose the lab equipment to test for Covid-19 infections (instead of e.g. controlled substances), and reluctance of police officers to submit evidence that might have come in contact with Covid-19 or to travel to the lab in general.

20 Some evidence suggests that police officers indeed made less arrests during the early days of the Covid-19 pandemic (see Rapisarda et al., 2020). However, it is possible that this was an intentional policy—aimed to reduce the crowding of jail houses.

21 Bullinger et al. (2020) find some mixed evidence regarding reporting behavior during the Covid-19 pandemic in Chicago: neighbors were found to be more likely to report domestic crimes because they spent more time at home. Conversely, victims were less likely to report.

22 The evidence from the Covid-19 pandemic suggests that indeed prisons are an epicenter (see, e.g., Akiyama et al., 2020).

23 There are also some countervailing evidence regarding the connection between prison conditions and crime. For instance, Chen and Shapiro (2007) and Drago et al. (2011) find that harsher conditions lead to more recidivism. It is unclear to which extent this would extend to the risk of being infected.

24 One potential solution for the job-finding aspect is “ban the box” policies, which forbids employers to pose a question on whether the applicant has a criminal record (see, e.g., Agan & Starr, 2018). However, this may lead to statistical discrimination, hurting minorities (Doleac & Hansen, 2020).

25 Of course, one could also engage in conspiracy via video-conferencing, but this increases the risk of being caught, as someone might try to record the video or hack into the conversation.

26 This would be in line with the aforementioned findings by Abrams (2021), where non-residential burglaries increased in the early days of the pandemic whereas residential burglaries decreased.

27 Of course, one should avoid double-counting, i.e. any effect that is already counted on the side of the payoff from crime should not be considered also on the side of the payoff from innocence.

28 Another explanation is an incapacitation effect: teenagers are at schools and thus unavailable for committing crimes (see, e.g., Machin et al., 2012).

29 One exception is a situation when the risk of a wrongful conviction is completely independent of the decision on whether to commit a crime (see Lando, 2006). However, note that the effect of wrongful convictions on crime is a matter of ongoing scholarly debate (see Garoupa & Rizzolli, 2012; Polinsky & Shavell, 2007; Lando & Mungan, 2017), and many papers follow the assumption that wrongful convictions do lower deterrence (see Pyne, 2019).

30 These points, and others, constitutes aspects measured in the increasingly popular database on exonerations in the U.S—the national registry of exoneration. The analyses conducted thus far confirm a connection between these and the probability of exoneration, where exonerations can serve as a proxy for wrongful conviction (see Gross & Shaffer, 2012; Sarel, 2018).

31 Bošković and Nenadić (2021, pp. 475) notes that during Covid-19, Portugal restricted the access of prisoners to their lawyers.

32 The theory is consistent with empirical evidence (Sarel, 2018), but also with experimental evidence showing that judges trade-off convictions and sentences (Feess et al., 2018, finding that higher fines reduce judge’s punishment frequency if and only if there is legal uncertainty).

33 Mungan (2017a) also shows how increases in the probability of apprehension indirectly leads to more aggregate deterrence when there are escalating punishments for repeat offenders.

34 The dashed line presents all possible expected utilities, depending on what *p* is.

35 Note that even with risk-neutrality, a social planner might prefer to maximize the penalty *s* and set a low *p*, as increasing *p* is typically costly. However, with risk-aversion, a maximal *s* is optimal even when changing *p* is costless.

36 For papers challenging Becker’s conclusion regarding risk attitudes, see Anderson et al. (2017) and Mungan (2017a).

37 For simplicity, ignore wrongful convictions here. Note that in order to remain consistent with the previous example, the sanctions are calculated here again as the additional reductions of wealth above and beyond the stolen money.

38 One particularly complex possibility would arise if reference points are expectation-based (see Kőszegi & Rabin, 2006; Feess & Sarel, 2021), i.e. individuals neither consider the status quo as their benchmark nor some previous point in time. Instead, the expected payoffs (taking into account the sanction as well) would serve as the benchmark and deviations would yield some loss/gain utility component. Speculating how such reference points would affect the decision to commit a crime, and in particular whether one could use them to improve deterrence (see Van Winden & Ash, 2012), is better left for future research.

39 Rizzolli and Stanca (2012) show that loss aversion affects also the payoff from wrongful convictions, which changes the opportunity cost of crime. Nicita and Rizzolli (2014) further consider loss aversion and extend the analysis to additional divergences from expected utility thoery, such as rank-dependent utility and emotions.

40 Note that the logic extends also to goods with higher value than supplies and for which supply chains were disrupted, such as cars (see, e.g., Belhadi et al., 2021).

41 Note that Becker (1968, pp. 18) already showed that if individuals are risk-seeking, then deterrence is maximized “by selecting positive and finite values” of the sanction and the probability of punishment.

42 For instance, the expected sanction is $$\delta *p*s$$, where $$0 \le \delta \le 1$$.

43 Another reason for discounting is, for instance, the fact that future payoffs are risky. In the Becker model, this is captured by the probability *p* anyway.

44 This concern does not apply to countries where medical treatment is subsidized by the state.

45 One reason for misestimations may be behavioral biases and heuristics (for a general overview, see Tversky & Kahneman (1974); For a discussion of how biases may affect behavior during the Covid-19 pandemic, see Teichman & Underhill (2021)).

46 For discussions of substitution effects in criminal behavior, see, e.g., Kahan (1996) and Elm and Sarel (2021). Note that one must first define what “deterrence” means: if deterrence is simply discouraging a particular behavior, then a switch to another harmful behavior still implies that deterrence works. However, if one takes a holistic view and considers what happens to the overall level of criminal activity, then substitution effects indeed mean that deterrence may not change.

47 Ma and McKinnon (2021) argue that Covid-19 has created new opportunities for cyber criminals, as people work from home and are more vulnerable to a wide range of crimes (scams, hacks, etc.).

48 The controversy is reflected, for example, in a series of comments on the earlier paper by Donohue & Levitt (Joyce, 2006; Foote & Goetz, 2008) and responses by the authors (Donohue & Levitt, 2004, 2008).

49 Some US governors even declared that abortions should cease during Covid-19 (see Bayefsky et al., 2020).

50 This saying is traditionally attributed to Socrates (see Dillon, 1979).
